# Comparison of Pooled Risk Estimates for Adverse Effects from Different Observational Study Designs: Methodological Overview

**DOI:** 10.1371/journal.pone.0071813

**Published:** 2013-08-20

**Authors:** Su Golder, Yoon K. Loke, Martin Bland

**Affiliations:** 1 Centre for Reviews and Dissemination (CRD), University of York, York, United Kingdom; 2 Norwich Medical School, University of East Anglia, Norwich, United Kingdom; 3 Department of Health Sciences, University of York, York, United Kingdom; Copenhagen University Hospital Gentofte, Denmark

## Abstract

**Background:**

A diverse range of study designs (e.g. case-control or cohort) are used in the evaluation of adverse effects. We aimed to ascertain whether the risk estimates from meta-analyses of case-control studies differ from that of other study designs.

**Methods:**

Searches were carried out in 10 databases in addition to reference checking, contacting experts, and handsearching key journals and conference proceedings. Studies were included where a pooled relative measure of an adverse effect (odds ratio or risk ratio) from case-control studies could be directly compared with the pooled estimate for the same adverse effect arising from other types of observational studies.

**Results:**

We included 82 meta-analyses. Pooled estimates of harm from the different study designs had 95% confidence intervals that overlapped in 78/82 instances (95%). Of the 23 cases of discrepant findings (significant harm identified in meta-analysis of one type of study design, but not with the other study design), 16 (70%) stemmed from significantly elevated pooled estimates from case-control studies. There was associated evidence of funnel plot asymmetry consistent with higher risk estimates from case-control studies. On average, cohort or cross-sectional studies yielded pooled odds ratios 0.94 (95% CI 0.88–1.00) times lower than that from case-control studies.

**Interpretation:**

Empirical evidence from this overview indicates that meta-analysis of case-control studies tend to give slightly higher estimates of harm as compared to meta-analyses of other observational studies. However it is impossible to rule out potential confounding from differences in drug dose, duration and populations when comparing between study designs.

## Introduction

A variety of study designs (including randomized controlled trials and observational studies) are used in the evaluation of adverse effects, and data from these diverse sources may be incorporated into subsequent systematic reviews and meta-analyses [1]. However, it is unclear whether differences amongst the study designs may contribute to discrepant estimates of harm that varies with the type of study. While there has been considerable debate regarding the pros and cons of evaluating adverse effects with non-randomised studies [2], [3], a recent methodological overview found that meta-analyses of observational studies yielded estimates of harm similar to those from randomized controlled trials [4]. Even then, methodological variation amongst the diverse categories of observational studies (such as cohort or case-control studies) could potentially lead to different estimates and inferences about adverse effects [5]. Case-control studies are often considered lower in the hierarchy of evidence compared to cohort studies, but are widely used in assessing rare harms [2], [6]–[11]. However, case-control studies do have potential biases stemming from ascertainment of exposure [12] that may lead to divergent findings compared to studies that use other methods [7], [13]–[17].

The extent of any discrepancy or heterogeneity between the pooled risk estimates from case-control studies and other study designs is a key concern for systematic reviewers. Previous research has tended to focus on differences in beneficial effects [18]–[24] or the differences in adverse effects between RCTs and observational studies [4]. There is some indication from our recent overview that case-control studies may potentially give higher estimates of harm compared to RCTs, whereas cohort studies seem to give similar estimates as the RCTs [4]. However, this overview was based on a relatively limited number of meta-analyses, and differences between observational designs were not formally evaluated. Hence, we aimed to explore the concordance between pooled estimates of the risk of adverse effects from case-control studies compared to pooled estimates from other observational designs.

## Methods

### Search Strategy

To identify studies for inclusion searches were undertaken in 10 key electronic databases to retrieve methodological papers related to any aspect of the incorporation of adverse effects into systematic reviews. These databases were carefully selected to allow the identification of reports, dissertations, and grey literature in addition to journal articles. A list of the databases and other sources searched is given in Appendix S1, Box 1. In addition, the bibliographies of any eligible articles identified were checked for additional references and citation searches were carried out for all included references using ISI Web of Knowledge. The search strategy used to identify relevant methodological studies in the Cochrane Methodology Register is described in full in Appendix S1, Box 2. The searches were not restricted to any particular adverse effect. This strategy was translated as appropriate for the other databases. No language restrictions were applied to the search strategies. However, due to logistical constraints only non-English papers for which a translation was readily available were retrieved.

Due to the difficulty of searching for methodological papers we also undertook handsearching of selected key journals, conference proceedings and web sources, and made contact with other researchers in the field. In particular, one reviewer (SG) undertook a detailed handsearch focusing on the Cochrane Database of Systematic Reviews (CDSR), and Database of Abstracts of Reviews of Effects (DARE) to identify systematic reviews that had evaluated adverse effects as a primary outcome and had a methodological analysis embedded [25]. A second reviewer (YKL) checked the included and excluded papers that arose from this handsearch.

### Eligibility Criteria

A meta-analysis or methodological evaluation was considered eligible for inclusion in our overview if it included case-control studies and at least one other type of observational study design (for example, cohort studies or cross-sectional studies) in the identification and/or quantification of an adverse effect or effects of a health-care intervention. Any healthcare intervention was deemed to be relevant including pharmaceutical interventions, diagnostic procedures, surgical interventions and medical devices. Relevant articles had to provide pooled estimates of the risk (risk ratio or odds ratio) of adverse effects according to different observational study designs.

Articles were independently assessed by two reviewers for potential included studies (S.G. and Y.K.L.). Full copies of the articles which were deemed potentially relevant by either reviewer were obtained. These articles were then reassessed and consensus reached after discussion.

### Data Extraction

Information was extracted on, the primary objective of the meta-analysis or methodological evaluation, included study designs, interventions and adverse effects evaluated. The number of primary studies included in the pooled analysis, number of patients by study design and the number of adverse effects observed in the treatment and control arm or comparator group were also recorded as were the type of summary statistic used in assessing differences between studies. We relied on the categorisation of study design as specified by the author of the meta-analysis or methodological evaluation. For example, if the authors stated that they looked at case-control studies and cohort studies, it was assumed that the studies were indeed case-control studies and cohort studies.

### Assessing the Validity of Comparing Pooled Estimates from Different Sets of Studies

The following criteria were used to consider the validity of comparing risk estimates across different study designs;

1. Presence of other factors that may have accounted for variation in results between studies of different designs
2. Discrepancies between the results obtained from different study designs may arise because of confounding factors other than study design (such as differences in population, delivery of intervention, or outcome measurement). A record was made of whether the authors of the meta-analysis or methodological evaluation reported that they had checked if the groups of different studies shared similar features in terms of population, interventions, comparators, and measurement of outcomes.
3. Heterogeneity in the pooled estimates
4. A record was made of whether the authors of the meta-analysis or methodological evaluation explored heterogeneity amongst the primary studies (using measures such as Chi^2^ or I^2^). An indication of heterogeneity of each set of pooled estimates by study design was assessed using a cut-off point of P<0.10 for Chi^2^ test results and 50% for I^2^ results [26]. In the few instances where both statistics were presented, the results of the I^2^ test were given precedence [27].
5. Statistical analysis comparing pooled estimates from study designs
6. A record was made of whether the authors of the meta-analysis or methodological evaluation described the statistical methods by which the magnitude of the difference between pooled estimates from different study designs was assessed.

Validity assessment and data extraction were carried out by one reviewer (S.G.) and checked by a second reviewer (Y.K.L.). All discrepancies were resolved after going back to the original source papers, with full consensus reached after discussion.

### Data Analysis

We checked for potential discrepancy between the pooled odds ratios (OR) from meta-analyses of different study designs by (i) quantitatively and graphically comparing the ratio of the pooled odds ratios from each study design, and (ii) comparing the separate point estimates and overlap in confidence intervals. Because adverse effects are rare, ORs and RRs were treated as equivalent [28].

In order to quantitatively describe the extent of discrepancy between study designs, we calculated the ratio of odds ratios (ROR) by taking the pooled OR for the adverse outcome from one study design divided by the pooled OR for the adverse outcome from another study design. If the meta-analysis of one study design for a particular adverse effect yielded exactly the same OR as the meta-analysis of another study design (i.e. complete agreement, or no discrepancy between study designs), then the ROR would be 1.0 (and Ln ROR = 0) [4], [29]. In order to maintain consistency in the direction of effect, pooled odds ratios from case-control studies were considered as the comparator (or denominator), which means that ROR <1 are indicative of cohort/cross-sectional studies giving lower estimates of harm compared to case-control studies. Conversely ROR >1 indicate that case-control studies have yielded lower odds ratios than the other observational designs.

The estimated RORs from each ‘cohort vs. case-control study’ comparison were then combined in a meta-analysis (random effects inverse variance method [30] – RevMan 5.1) to summarize the overall ROR between cohort and case-control studies across all the included reviews. The standard error (SE) of ROR can be estimated using the standard errors for the case-control study and other observational design estimates respectively:
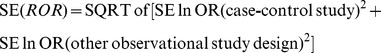



Standard errors pertaining to each pooled OR(case-control study) and OR(other observational study design) were calculated from the published 95% CI [31]. Statistical heterogeneity was assessed using *I^2^* statistic, with *I* ^2^ values of 30–60% representing a moderate level of heterogeneity [32].

Funnel plots were constructed to evaluate the distribution of the ROR against estimates of precision (1/SE) [30], [33]. If there were no systematic differences or discrepancies between the pooled OR from the various study designs, we would expect the ROR data points to be symmetrically distributed within the funnel shape. Conversely, if one set of study designs consistently generated either lower or higher risk estimates, then the RORs would be skewed to one side, with an asymmetrical funnel plot.

We also provide a descriptive summary of the data in terms of confidence interval (CI) overlap between pooled sets of results by study design, and any differences in the direction of effect between study designs. The results were said to agree if both study designs identified a significant increase, a significant decrease or no significant difference in the adverse effects under investigation.

## Results

### Included Studies

**Figure 1:**
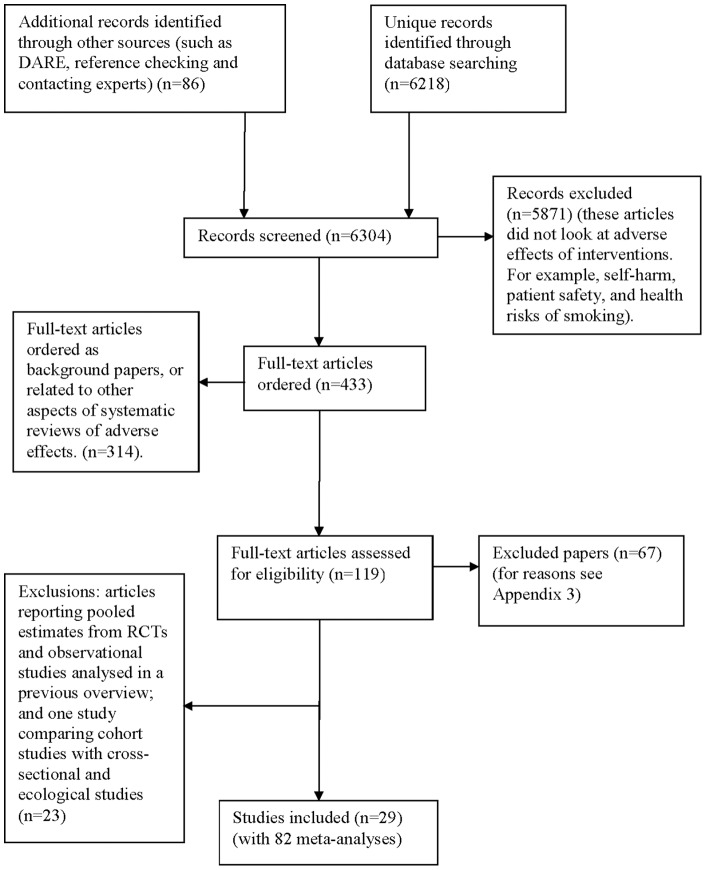
Flow chart for included studies.

6218 unique records were retrieved from the electronic database searches and 86 records from additional sources (such as DARE, reference checking and contacting experts) (Figure 1). In total 433 full papers were retrieved. 314 articles were excluded as they were ordered as background papers or related to other aspects of systematic reviewing. Appendix S3 lists 67 articles that were excluded from our methodological overview during the screening and data extraction phases, with the reasons for exclusion.

A further 22 articles compared RCTs with observational studies (in some instances using the incidence of adverse effects – without reporting RR/ORs) [34]–[56], and one more compared cohort studies with cross-sectional studies and ecological studies but did not include case-control studies [57]. Studies with pooled estimates of RCT and observational data on adverse effects have been analysed and reported separately in another manuscript [4].

We therefore included 29 eligible articles which contained a total of 82 meta-analyses where it was possible to compare the pooled risk estimates from case-control studies against that of cohort or cross-sectional studies. Some articles included more than one meta-analysis, usually for the same adverse effect but with slight variations in the intervention, for example with different drug dosages, exposure times or different drugs within the same class. The 82 meta-analyses included a total of 521 case-control studies, 302 cohort studies, and 38 cross-sectional studies (Appendix S2, table 1).

**Table 1:** Ratio of odds ratios of adverse effects in study design comparisons.

Comparison of meta-analyses according to study design	Overall ratio of odds ratios (RORs)	Heterogeneity
Cohort versus case-control (n = 64)	0.94 (95% CI 0.87 to 1.01)	I^2^ = 55%
Cross-sectional versus case-control studies (n = 18)	0.92 (95% CI 0.81 to 1.05).	I^2^ = 24%
Cohort and cross-sectional versus case-control (n = 82)	0.94 (95% CI 0.88–1.00)	I^2^ = 55%

Only one of the 29 articles was a methodological evaluation with the primary aim to assess the impact of study design [58], whereas the remaining 28 were systematic reviews within which results of subgroup analysis by study design was embedded.

### Interventions

Most (27/29, 93%) focused on adverse effects of pharmacological interventions (such as oral contraceptives, NSAIDs, or HRT) [58]–[71], [72], [73]–[84]. Other topics assessed were a surgical intervention (caesarean delivery) [85], and a diagnostic test (ultrasonography) [52].

### Assessing the Validity of Comparing Pooled Estimates from Different Sets of Studies

#### 1. Presence of other factors that may have accounted for variation in results between studies of different designs

Although many of the methodological evaluations acknowledged the potential for confounding factors that might yield discrepant findings between study designs, no adjustment for confounding factors was reported in most instances [52], [59]–[64], [67]–[85]. There were three instances where the authors of the methodological evaluations performed some adjustment for potential confounding factors [58], [65], [66]. Two carried out meta-regression [58], [65], and one measured differences in heterogeneity between study designs [66]. In two of the methodological evaluations, other factors (such as drug dose and duration) were thought to be potentially responsible for discrepancies across the different study designs [65], [66]. In addition, a few authors carried out subgroup analysis stratified for factors such as population characteristics, drug dose, or duration of drug exposure which may help increase the similarity of the pooled studies being compared.

#### 2. Heterogeneity in the pooled estimates

12 reviews measured the heterogeneity of at least one set of the included studies grouped by study design using statistical analysis such as Chi^2^ or I^2^ [52], [58], [61]–[63], [67], [68], [73], [75], [79]–[81]. Case-controlled studies were more likely to exhibit heterogeneity than cohort studies, with 16/19 (84%) of the pooled sets of case-control studies showing evidence of heterogeneity [61], [62], [63], [67], [73], [79]–[81], whereas only 6/18 (33%) of the pooled sets of cohort studies experienced significant heterogeneity [52], [58], [61], [67], [80].

#### 3. Statistical analysis comparing pooled estimates from different study designs

Authors of four reviews explicitly tested for a difference between the results by study design using p-values [61], [65], [68], [85]. Three reviews reported on the heterogeneity of the pooled studies of one design, the pooled studies of another design, and of all the studies combined [61], [68], [80]. This can indicate statistical differences where the pooled study designs combined are significantly heterogeneous but no significant heterogeneity is seen when the study designs are pooled separately.

### Data Analysis

Appendix S4 documents the decisions made in instances where the same data were available in more than one format.

#### Pooled analysis of the ROR estimates

The calculated differences between study designs (ROR) for each adverse effect were summarized together in a random effects model to give an average picture of the extent of discrepancy (Table 1 and Appendix S5). On average, cohort studies yielded pooled odds ratios 0.94 (95% CI 0.87–1.01) times lower than that from case-control studies, whereas cross-sectional studies had pooled odds ratios 0.92 (0.81–1.05) times lower than that from case-control studies).

Overall, the pooled ROR of 0.94 (95% CI 0.88–1.00) from the study design comparisons shows that on average, meta-analyses of meta-analyses of cohort and cross-sectional designs gave odds ratios that were a relative 6% lower than those from meta-analyses of case-control studies. Although the differences between study designs did not reach conventional threshold of statistical significance, the low to moderate heterogeneity seen overall is an indicator that there may be consistent pattern of variation between study designs.

#### Funnel Plots: ROR from cohort and cross-sectional studies versus case-control studies

**Figure 2:**
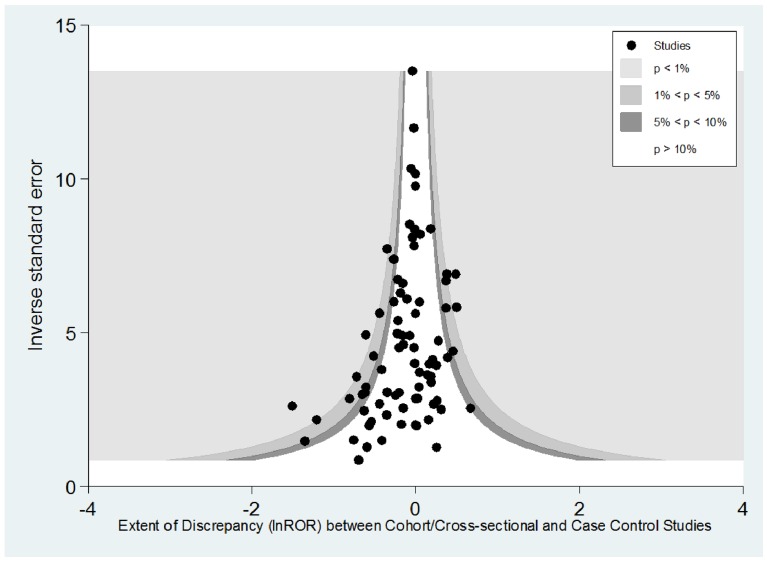
Funnel plot of distribution of RORs from meta-analyses of cohort/cross-sectional studies compared to case-control studies.

Visual inspection of the Funnel Plot (Figure 2) and results from the Egger test (p = 0.02) suggests that there is an asymmetrical distribution of the discrepancy between study designs (RORs) and that this asymmetry is significant statistically. There seem to be fewer instances where meta-analyses of case-control studies gave lower estimates of harm, and a relative predominance of studies on the left side of the plot showing that case-control studies frequently tended to give higher estimates of risk than those from cohort or cross-sectional studies. The shape of this funnel plot would be consistent with the overall ROR estimate of 0.94 (0.88–1.00) described in Table 1. In pooled estimates with greater precision (located at upper end of the funnel plot), there did not appear to be much discrepancy between the study designs. The shape of the funnel plot was similar when we individually compared pooled estimates from cohort studies, or cross-sectional studies against case-control studies (Appendix S6).

### Confidence Interval Overlap

The confidence intervals for all the pooled results from case-control studies and cross-sectional studies overlapped, and almost all the confidence intervals (CIs) for the pooled results from case-control studies and cohort studies overlapped (94%, 60/64) (Table 2).

**Table 2:** Confidence interval overlap and agreement between study designs.

Study design comparisons	Number of instances of confidence interval overlap	Agreement in findings between the study designs			Discrepancy in findings between the study designs		
		Both showed a significant increase in adverse effect with intervention	Both did not identify any significant difference in adverse effect between intervention and control	Both showed a significant decrease in adverse effect with intervention	Significant risk increase in one vs. significant risk decrease in the other	Significant increase in one vs. no significant difference in the other	Significant decrease in one vs. no difference in the other
Cohort studies versus Case-control. (n = 64)	60 (94%)	19 (27%)	23 (38%)	0	1 (2%) (one showed decrease in adverse effect in cohort studies but increase in case-control studies)	20 adverse effects (31%) with significant ↑ risk (seen with 14 meta-analyses of case-control studies, and 6 meta-analyses of cohort studies)	1 (2%) (One showed decrease in adverse effects with cohort studies but no difference in case-control studies)
Cross-sectional studies versus Case-control (n = 18)	18 (100%)	4 (22%)	11 (61%)	0	0	3 adverse effects (17%) with significant ↑ risk (seen with 2 meta-analyses of case-control studies, one meta-analysis of cross-sectional studies)	0

### Agreement and Disagreement of Results

#### Agreement in results

In most of the reviews the results of the adverse effects estimates agreed between types of study design [52], [58]–[60], [63], [65]–[67], [69]–[84]. Most reviews that demonstrated an agreement between study designs did not find a significant increase or significant decrease in the adverse effects under investigation (Table 2).

The tendency for case-control studies to show greater degree of harm is illustrated by the 16 adverse effects where meta-analyses of case-control studies found significantly elevated risk, but meta-analyses of cohort or cross-sectional studies did not confirm this risk. Conversely, there were 7 adverse effects where meta-analyses of cohort or cross-sectional studies demonstrated significantly elevated risk, but meta-analyses of case-control studies did not show significant risk.

#### Disagreement in Conclusions

There was one major discrepancy in one pooled sets of results. Grady et al 1995 [66] found that whilst cohort studies demonstrated a decrease in endometrial cancer (or protective effect) with estrogen plus progestin (RR 0.4 (0.2–0.6)), case-control studies demonstrated an increase (1.8 (1.1–3.1)).

#### Sensitivity analysis: limited to one review per adverse effect examined

There are no adverse effects where two or more separate meta-analyses have used exactly the same primary studies, (i.e. had complete overlap of case-control and cohort studies) to generate the pooled estimates. This reflects the different time periods, varying search strategies and inclusion and exclusion criteria that have been used by authors of these meta-analyses such that even though they were looking at the same adverse effect, they used data from different studies in generating pooled overall estimates.

There were three adverse effects that were evaluated in more than one review; venous thromboembolism (VTE), gastrointestinal complications and stroke. In the few instances where adverse effects were described in more than one review, sensitivity analysis limiting it to one review per adverse effect showed similar ROR (Appendix S7).

## Discussion

We found that on average, meta-analysis of case-control studies tended to give slightly higher estimates of harm as compared to cohort or cross-sectional studies. This finding was reflected in the asymmetrical shape of the funnel plot showing that the direction of the discrepancies (as estimated by the RORs) were more frequently due to relatively higher estimates of harm from case-control studies. Alternatively, this could be interpreted as cohort studies being more susceptible to underestimating the extent of harm. This is illustrated by our finding of 16 adverse effects where case-control studies showed significant harm but cohorts and case-controls did not. Conversely there were seven adverse effects where the opposite was true, with case-control studies showing no increase in risk, whereas the other observational designs found significant likelihood of harm. Given that observational study designs are important data sources on rare adverse effects, we recommend that readers of medical journals, as well as systematic reviewers should evaluate consistency of findings across a broad range of study designs when considering rare harms.

### Potential Reasons for the Discrepancy between Study Designs

An explanation for the tendency for slightly higher estimates of harm from case-control studies is difficult to ascertain. However, there are a number of possible reasons. Firstly, this could be a spurious result as the values for the ROR do not reach statistical significance. Nevertheless, the asymmetrical funnel plot does demonstrate a fairly consistent discrepancy between cohort studies compared to case-control studies. One important factor here may stem from the potentially greater ability of case-control studies to enrol sufficient numbers of patients who are known to have experienced a rare adverse event, thus yielding more statistical power to detect small, but significant risk of harm [86]. Another reason could be related to differences in susceptibility to bias amongst study designs, where bias in case-control studies may arise if cases and controls do not have equal opportunity for past exposure (or if ascertainment of exposure is biased) [62]. Nevertheless, case-control studies based on pharmacoepidemiological databases with pharmacy and medical record linkage may not be susceptible to such recall bias. Conversely, bias in cohort studies can develop if the exposed and unexposed groups do not have equal opportunity for having the adverse event to happen (or to be measured) and doctors may be more likely to undertake diagnostic investigations or recommend more frequent follow-up in patients taking certain types of medications [61].

Equally, discrepancies between study designs could have stemmed from confounding as a result of variation in characteristics of participants, timing and site of study, and definitions of exposure and outcomes. For instance, if one set of studies are carried out on a younger cohort of patients, with a lower drug dosage, or with shorter duration of use, or relied on passive ascertainment of adverse effects data [10], [48], [87], [88], it might be expected that the magnitude of any adverse effects recorded would be lower. However, most of the evaluations were not conducted with the primary aim of assessing differences in study design but were systematic reviews with some secondary comparative evaluation of study design embedded within them. It is not surprising, therefore, that many did not consider confounding factors. In many instances, it may also not have been possible to control for numerous potential confounding factors as the primary studies may not have contained the required information. The small number of studies included (sometimes as low as one) may have not enabled statistical analysis such as meta-regression to be undertaken. Nevertheless, the asymmetrical pattern of the funnel plot would tend to suggest a more systematic cause of discrepancy between study designs, rather than just chance variation in participants and definitions of exposure and outcome. The design of case-control studies may involve a greater extent of selection of risk factors for analysis and reporting, and significant findings may be more likely to be selectively published (and thus subsequently included in systematic reviews).

Finally, differences in observed and unobserved patient characteristics may have accounted for discrepancies between designs. The extent of statistical adjustment for potential confounders in observational studies depends somewhat on which variables were measured in the primary dataset. Given the different starting points in data collection between case-control and cohort studies, the effect of unmeasured confounders may afflict either design to dissimilar extents.

### Comparisons to other studies

Our previous work showed that meta-analyses of RCTs versus cohort studies showed little discrepancy ROR 1.02 (95% CI 0.82–1.28) whereas meta-analyses of RCTs versus case-controls showed a greater discrepancy with a ROR of 0.84 (95% CI 0.57–1.23), thus indicating that case-control studies give higher estimates of harm when compared to RCTs. Given that cohort studies showed similar risk of harm compared to RCTs, it can be indirectly inferred that case-control studies also provide a higher risk of harm when compared to cohorts. Our current analysis would be entirely consistent with the previous findings.

## Limitations

Our overview was constrained by information and data contained in the included evaluations as it was not straightforward to source and evaluate the >850 primary studies contained in the meta-analyses. In each instance the authors’ categorisation of the study design was used. However, we note that most of the included reviews had passed DARE criteria or were from peer-reviewed sources i.e. both the primary study and the systematic review had undergone peer-review. Moreover, any misclassification is likely to be non-differential in impact, which should not lead to elevated risk estimates from any particular study designs.

Another important limitation to this review is the potentially unrepresentative sample used. Systematic reviews with embedded data comparing different study designs may have been missed. The search strategy used was limited to a literature search to identify methodological papers whose primary aim was to assess the influence of study design and to a sift of systematic reviews of adverse effects identified from the Cochrane Database of Systematic Reviews (CDSR) and Database of Abstracts of Reviews of Effects (DARE). Nevertheless, it should be noted that the CDSR and DARE databases cover a large proportion of all systematic reviews and that systematic reviews in which adverse effects are included as a secondary aim are unlikely to present subgroup analysis by study design for the adverse effects data.

There was considerable heterogeneity between the comparisons of different studies, suggesting that any differences could be specific to particular types of interventions or adverse effects. It may be that particular types of adverse effects can be identified more easily via particular types of study designs [5], [89]–[91]. However, it was difficult to assess the evaluations by type of adverse effects (such as long-term or rare). This would be of interest, given that the literature suggests that RCTs may be better at identifying some types of adverse effects (such as common, anticipated and short-term) more effectively than observational studies.

### Implications for clinical practice and research

In the light of our findings, we believe that regulatory authorities, as well as interested patients and physicians, who appraise articles on adverse effects, should look carefully at the study designs involved, and be aware of potential differences in whether the particular design may tend to provide relatively higher or lower estimates on risk of harm. The differences between study designs are most apparent when the meta-analysis only has a few studies, thus suggesting that we should be particularly cautious in trusting single studies of rare harms. Further research should evaluate the impact of different study designs across a wide range of adverse effects in multiple databases. As an example, Ryan et al 2012^85^ have already looked at methods of signal generation for detecting new adverse events in 10 observational databases, and extending this approach to signal refinement or hypothesis testing should clearly be feasible.

Our overview also has important implications for the conduct of systematic reviews of harm, particularly with regards to selection of a broad range of relevant studies. Although there are strengths and weaknesses to each study design, empirical evidence from this overview indicates that there are slight differences (on average) between estimates on the risk of adverse effects obtained from meta-analyses of different observational study designs. Instead of restricting the adverse effects analysis to certain study designs (which might lead to a potentially one-sided view), it seems preferable for systematic reviewers to evaluate a broad range of studies that can help build a complete picture of any potential harm and improve the generalisability of the review without loss of validity.

## Supporting Information

Appendix S1 **Search strategy and Data Sources.**
(DOCX)

Appendix S2 **Characteristics of Included Studies.**
(DOCX)

Appendix S3 **Excluded Studies and Reasons for Exclusion.**
(DOCX)

Appendix S4 **Selection of outcomes and handling of duplicate data.**
(DOCX)

Appendix S5 **Forest Plot: meta-analysis of RORs from cohort/cross-sectional studies versus case-control studies.**
(DOCX)

Appendix S6 **Funnel plot of distribution of RORs from (i) meta-analyses of cohort studies compared to case-control studies (ii) meta-analyses of cross-sectional studies compared to case-control studies.**
(DOCX)

Appendix S7 **Sensitivity analysis based on exclusion of meta-analyses covering the same adverse effect where there is some overlap in the included primary studies.**
(DOCX)
