# Supplementary material for: Comparison of Pooled Risk Estimates for Adverse Effects from Different Observational Study Designs: Methodological Overview
Source: PLoS One. 2013 Aug 20;8(8):e71813. doi: 10.1371/journal.pone.0071813 (PMC3748094; doi:10.1371/journal.pone.0071813)
Supplement: Appendix S1 — Search strategy and Data Sources. (DOCX) [file pone.0071813.s001.docx]

**Appendix 1**

**Box 1: Sources searched for included studies**

*Databases*

All databases were originally searched on the 26^th^ or 27^th^ September 2007 with the most recent update searches carried out between the 22^nd^ October and 6^th^ November 2009.

Cochrane Database of Systematic Reviews (CDSR): methodology reviews only: 2009 Issue 4

Cochrane Methodology Register (CMR): 2009 Issue 4

Database of Abstracts of Reviews of Effects (DARE): CRD Internal Database$: November 2009

EMBASE: 1980 to 2009 Week 42

Health Technology Assessment (HTA) Database: November 2009

Health Management Information Consortium (HMIC): September 2009

Index to Theses: November 2009

Library, Information Science & Technology Abstracts (LISTA): Mid-1960s – October 2009

MEDLINE: 1950 to October Week 3

MEDLINE in process: 22 October 2009

*Handsearching of Journals*

BMC Clinical Pharmacology - 2001;1(1) to 2009;9(17)

BMC Medical Research Methodology - 2001;1 to 2009;9(69)

Drug Safety – 1998;18(1) to 2009;32(11)

Health Information and Libraries Journal (formerly Health Libraries Review) - 1994;11(1) to 2009;26(3)

Journal of Clinical Epidemiology - 1998 to 2009;62(12)

Journal of Information Science - 1979;1(1) to 2009;35(5)

Journal of Librarianship and Information Science - 1969;1(1) to 2009;41(3)

Journal of the Medical Library Association (formerly the Bulletin of the Medical Library Association) - from 2000;88(2) to 2009;97(4)

Pharmacoepidemiology & Drug Safety – 1992;1(1) to 2009;18(11)

*Handsearching of Conference Proceedings*

Cochrane Colloquia 1994 - 2009

HTAi 2004 - 2009

Pharma-Bio-Med Conference and Exposition 2006 - 2008

Symposium on Systematic Reviews 1998 - 2002

*Web Sources*

Agency for Healthcare Research and Quality (AHRQ) via <http://www.ahrq.gov/> Searched: 28/10/09

Health Technology Assessment Programme (HTA) via <http://www.hta.ac.uk/index.shtml>

Searched: 28/10/09

$ The version of the DARE database searched was the internal CRD administration database which contains all the included systematic reviews as well as other papers retrieved in the search process for systematic reviews including methodological papers. The DARE database is compiled through searches of MEDLINE, EMBASE, PubMed, PsycINFO, CINAHL, AMED and ERIC and handsearching of key journals, grey literature and regular searches of the Internet.

**Box 2: Example search strategy**

**Cochrane Methodology Register (CMR)**

Interface: http://www.thecochrane library.com

Version: 2007 Issue 3 (original search), 2008 Issue 3 (first update search), 2009 Issue 4 (second update search)

Date Searched: 26/09/07 (original search), 17/08/09 (first update search), 22/10/09 (second update search)

The following search strategy, using terms in the title, abstract and keywords, retrieved 1517 records in the original search, an extra 249 records in the first update search and 344 in the second update search;

#1 adverse

#2 side next effect*

#3 unintended next effect*

#4 unintended next event*

#5 unintended next outcome*

#6 unintended next reaction*

#7 unintended next interaction*

#8 unintended next response*

#9 unintentional next effect*

#10 unintentional next event*

#11 unintentional next outcome*

#12 unintentional next reaction*

#13 unintentional next interaction*

#14 unintentional next response*

#15 unwanted next effect*

#16 unwanted next event*

#17 unwanted next outcome*

#18 unwanted next reaction*

#19 unwanted next interaction*

#20 unwanted next response*

#21 unexpected next effect*

#22 unexpected next event*

#23 unexpected next outcome*

#24 unexpected next reaction*

#25 unexpected next interaction*

#26 unexpected next response*

#27 undesirable next effect*

#28 undesirable next event*

#29 undesirable next outcome*

#30 undesirable next reaction*

#31 undesirable next interaction*

#32 undesirable next response*

#33 adrs or ades or adr

#34 drug next surveillance

#35 post next marketing next surveillance

#36 postmarketing next surveillance

#37 treatment next emergent

#38 complication*

#39 tolerability

#40 toxicity

#41 harm or harms or harmful

#42 safety

#43 safe

#44 tolerance

#45 tolerate

#46 toxic

#47 risk or risks

#48 (#1 OR #2 OR #3 OR #4 OR #5 OR #6 OR #7 OR #8 OR #9 OR #10 OR #11 OR #12 OR #13 OR #14 OR #15 OR #16 OR #17 OR #18 OR #19 OR #20 OR #21 OR #22 OR #23 OR #24 OR #25 or #26 or #27 or #28 or #29 or #30 or #31 or #32 or #33 or #34 or #35 or #36 or #37 or #38 or #39 or #40 or #41 or #42 or #43 or #44 or #45 or #46 or #47)
