# Supplementary material for: Comparison of Pooled Risk Estimates for Adverse Effects from Different Observational Study Designs: Methodological Overview
Source: PLoS One. 2013 Aug 20;8(8):e71813. doi: 10.1371/journal.pone.0071813 (PMC3748094; doi:10.1371/journal.pone.0071813)
Supplement: Appendix S2 — Characteristics of Included Studies. (DOCX) [file pone.0071813.s002.docx]

**Appendix 2**

**Table 1: Characteristics of included studies**

| **Reference** | **Study Design** | **Included Studies** | **Methodological Assessment** | **Increase/decrease/no difference in adverse effects by study design** |
| --- | --- | --- | --- | --- |
| Agency for Healthcare Research and Quality 2002^1^ | Systematic review of hormone replacement therapy and venous thromboembolism. | ***Venous thromboembolism***  **1 cohort study (N=112593)**  RR 2.1 (1.2-3.8)  **8 case-control studies (N=23544)**  RR 2.05 (1.40-2.95) | **Confounding factors by study design:** NR  **Heterogeneity within study designs:** NR (No significant heterogeneity among all 12 studies P>0.10)  **Statistical analysis comparing study designs:** NR | **Cohort study:** Significant increase  **Case-control studies:** Significant increase  **CI overlap:** Yes |
| Bager et al 2008^2^ | Systematic review of caesarean delivery and atopy and allergic disease | ***Asthma***  **11 cohort studies (N=NR)**  OR 1.22 (1.09-1.37)  **2 case-control studies (N=NR)**  OR 0.84 (0.64-1.10) | **Confounding factors by study design:**  NR (Carries out stratified meta-analysis for adjustment of risk ratios, a priori aim, study design, year of birth, size of study population, country, exclusion, proportion of c-sections and age. For asthma significant variations were seen for age and study design. No further data shown)  **Heterogeneity within study designs:** NR (significant heterogeneity among all 13 studies P<0.01)  **Statistical analysis comparing study designs:** Higher ORs for cohort studies compared with case-control studies P<0.01. Summary OR variation with study characteristics, P-values two-tailed, based on likelihood ratio tests. | **Cohort studies:** Significant increase  **Case-control studies:** No significant difference  **CI overlap:** Yes |
| Bergendal et al 2009^3^ | Systematic review of progestogen-only contraception and venous thromboembolism | ***Venous thromboembolism***  **1 cohort study (N=204)**  OR 0.8 (0.2-3.9)  **4 case-control studies (N=10004)**  OR 1.45 (0.92-2.26) | **Confounding factors by study design:**  NR but did not include the cohort study in the meta-anlsys as deemed to deviate too much from other studies in terms of population and design.  **Heterogeneity within study designs:** NR  **Statistical analysis comparing study designs:** NR | **Cohort studies:** No significant difference  **Case-control studies:** No significant difference  **CI overlap:** Yes |
| Bollini et al 1992^4^ | Meta-analysis of NSAIDS and upper gastrointestinal tract disease with primary aim to assess the impact of study design and research quality. | ***Upper gastrointestinal tract disease***  **7 cohort studies (N=NR)**  RR 2.0 (1.2 to 3.2)  Chi^2^ P<0.01  **27 case-control studies (N=NR)**  RR 4.1 (3.2 to 5.3)** | **Confounding factors by study design:**  Stated that type of study design was independently associated with risk estimates, even after adjustment. Used multivariate regression to adjust in the same model for drug investigated, type of study design, and methodological quality.  **Heterogeneity within study designs:** No significant heterogeneity: (Community-based case-control studies N=8 Chi^2^ P>0.05).  Significant heterogeneity: (Hospital-based case-control studies N=19) and one set of cohort studies  **Statistical analysis comparing study designs:** NR but states that cohort studies significantly lower risk ratio estimate than hospital based case-control studies. | **Cohort studies:** Significant increase  **Case-control studies:** Significant increase  **CI overlap:** Yes |
| Capurso et al 2007^5^ | Systematic review of NSAIDS and pancreatic cancer. | ***Pancreatic cancer (low NSAID exposure)***  **3 Cohort studies (N=1,072,263)**  Aspirin/NSAIDS 883/244,404  Control 3668/827,859  OR 0.84 (0.64-1.09)  **3 Case-control studies (N=7,254)**  Aspirin/NSAIDS 347/3,302  Control 728/3,952  OR 1.04 (0.81-1.33)  ***Pancreatic cancer (IntermediateNSAID exposure)***  **3 Cohort studies (N=906,924)**  Aspirin/NSAIDS 363/79,065  Control 3,668/827,859  OR 0.94 (0.63-1.40)  **3 Case-control studies (N=4,648)**  Aspirin/NSAIDS 123/696  Control 728/3,952  OR 1.15 (0.69-1.91)  ***Pancreatic cancer (High NSAID exposure)***  **3 Cohort studies (N=851,932)**  Aspirin/NSAIDS 84/24,073  Control 3668/827,859  OR 0.94 (0.51-1.71)  **3 Case-control studies (N=4,267)**  Aspirin/NSAIDS 60/315  Control 728/3,952  OR 1.12 (0.52-2.41) | **Confounding factors by study design:** NR but conducts subgroup analysis by factors such as gender, aspirin use only, and nurse occupation.  **Heterogeneity within study designs:** NR (Significant heterogeneity among 7 studies with low exposure P=0.005, I^2^ =67.3%, 6 studies with intermediate exposure P=0.001, I^2^=75.0% and 6 studies with high exposure P<0.0001, I^2^=83.4%)  **Statistical analysis comparing study designs:** NR but states no significant difference. | ***Low exposure***  **Cohort studies:** No significant difference  **Case-control studies:** No significant difference  **CI overlap:** Yes  ***Intermediate exposure***  **Cohort studies:** No significant difference  **Case-control studies:** No significant difference  **CI overlap:** Yes  ***High exposure***  **Cohort studies:** No significant difference  **Case-control studies:** No significant difference  **CI overlap:** Yes |
| Chan et al 2004^6^ | Systematic review oral contraceptives and stroke. | ***Stroke***  **4 Cohort studies (N=>1,000,000)**  OR 0.95 (0.51-1.78)  Chi^2^ P=0.01  **16 Case-control studies (N=15,106)**  OR 2.13 (1.59-2.86)  Chi^2^ P<0.001 | **Confounding factors by study design:**  Authors comment on heterogeneity of studies, potential confounding and risk of bias e.g. cohort studies ‘might be methodologically superior .. present more valide assessment of stroke risk’.  **Heterogeneity within study designs:** Significant heterogeneity: One set cohort studies and one set of case control studies (and when all studies pooled)  **Statistical analysis comparing study designs:** Differences among subgroups were calculated using the standard gaussian Z statistic. The pooled odds ratio of the cohort studies was significantly different from that of the case-control studies P=0.03 | **Cohort studies:** No significant difference  **Case-control studies:** Significant increase  **CI overlap:** yes |
| Dolovich et al 1998^7^ | Systematic review of benzodiazepine use in pregnancy and major malformations and oral cleft. | ***Major malformations***  **7 cohort studies (N=72,866)**  Exposed 32/1090 Non-exposed 2783/71776  OR 0.90 (0.61-1.35)  Chi^2^ P=0.62  **4 case-control studies (N=6,136)**  Exposed 84/166 Non-exposed 2141/5970  OR 3.01 (1.32-6.84)  Chi^2^ P=0.008  ***Oral cleft***  **3 cohort studies (N=138,286)**  Exposed 1/2543 Non-exposed 93/135743  OR 1.19 (0.34-4.15)  Chi^2^ P=0.997  **6 case-control studies (N=14,971)**  Exposed 105/285 Non-exposed 2742/14686  OR 1.79 (1.13-2.82)  Chi^2^ P=0.01 | **Confounding factors by study design:**  Acknowledges systematic differences between study design e.g. exposure to other medications, duration and indication for use of benzodiazepine and possible differences in populations.  **Heterogeneity within study designs:** No significant heterogeneity: two sets of cohort studies.  Significant heterogeneity: two sets of case-control studies  **Statistical analysis comparing study designs:** NR | ***Major malformations***  **Cohort studies:** No significant difference P=0.62  **Case-control studies:** Significant increase P=0.008  **CI overlap:** yes  ***Oral cleft***  **Cohort studies:** No significant difference P=0.997  **Case-control studies:** Significant increase P=0.01  **CI overlap:** Yes |
| Douketis et al 1997^8^ | Systematic review of oral contraceptives and hormone replacement therapy and venous thromboembolism. | ***Venous thromboembolism (oral contraceptives)***  **7 cohort studies****  RR 3.0 (2.2-4.2)  prospective studies Chi^2^ P=0.8, retrospective studies Chi^2^ P=0.3  **12 case-control studies (N=NR)**  RR 3.0 (2.6-3.4)  Chi^2^ P=<0.001  ***Venous thromboembolism (Hormone replacement therapy)***  **1 cohort study (N=NR)**  RR 1.7 (1.0-2.9)  **5 case-control studies (N=NR)**  RR 2.4 (1.7-3.5)  Chi^2^ P=0.24 | **Confounding factors by study design:**  NR  **Heterogeneity within study designs:**  No significant heterogeneity: one set of case-control studies, (one set of prospective cohort studies  P=0.8 and one set of retrospective cohort studies P=0.3)  Significant heterogeneity: One set of case-control studies  **Statistical analysis comparing study designs:** NR | ***Oral contraceptives***  **Cohort studies:** Significant increase  **Case-control studies:** Significant increase  **CI overlap:** Yes  ***Hormone replacement therapy***  **Cohort studies:**  No significant difference  **Case-control studies:** Significant increase  **CI overlap:** Yes |
| Garg et al 1998^9^ | Systematic review of hormone replacement therapy and ovarian cancer. | ***Ovarian cancer***  **1 Cohort study (N=NR)**  RR 1.15 (0.94-1.42)  **9 Case-control studies (N=NR)**  RR 1.16 (1.03-1.29) | **Confounding factors by study design:**  NR  **Heterogeneity within study designs:** NR (no significant heterogeneity for all 10 studies P=0.72)  **Statistical analysis comparing study designs:** NR | **Cohort study:**  No significant difference  **Case-control studies:** Significant increase  **CI overlap:** Yes |
| Gillum et al 2000^10^ | Systematic review oral contraceptives and ischemic stroke. | ***Ischemic stroke***  **3 Cohort studies (1,069,840 person years)**  RR 3.21 (1.96-5.27)  **14 Case-control studies (N=9,920)**  RR 2.77 (2.22-3.45) | **Confounding factors by study design:**  Evaluated a number of potential confounders. Meta-regression analysis suggested estrogen dosage, control of smoking and firm diagnosis of ischemic stroke were the only study variables contributing to risk ratio estimates. Study design was not identified as contributing to risk ratio estimate.  **Heterogeneity within study designs:** NR (Significant heterogeneity among all studies P=0.01)  **Statistical analysis comparing study designs:** A 2 tailed z test was used to detect differences across subgroups but no p value was reported for study design. Authors state that similar positive associations found in case-control studies and cohort studies suggesting that this aspect of study design was unimportant. | **Cohort studies:** Significant increase  **Case-control studies**: Significant increase  C**I overlap:** Yes |
| Grady et al 1995^11^ | Systematic review of postmenopausal estrogen therapy and estrogen plus progestin and endometrial cancer . | ***Endometrial cancer*** (***Postmenopausal estrogen therapy)***  **4 Cohort studies (N=NR)**  RR 1.7 (1.3-2.1)  **25 Case-control studies (N=NR)**  RR 2.4 (2.2-2.6)  ***Endometrial cancer (Estrogen plus progestin)***  **2 Cohort studies (N=NR)**  RR 0.4 (0.2-0.6)  **3 Case-control studies (N=NR)**  RR 1.8 (1.1-3.1) | **Confounding factors by study design:**  Heterogenity substantially reduced or eliminated when the studies where stratified by dose or duration of estrogen use suggesting that these two variables account for most of the variation in risk estimatates.  **Heterogeneity within study designs:** NR (significant heterogeneity among all studies)  **Statistical analysis comparing study designs:** NR | ***Postmenopausal estrogen therapy***  **Cohort studies:**  Significant increase  **Case-control studies:**  Significant increase  **CI overlap:** No  ***Estrogen plus progestin***  **Cohort studies:**  Significant decrease  **Case-control studies:**  Significant increase  **CI overlap:** No |
| Henry and McGettigan 2003^12^ | Systematic review of NSAIDS and gastrointestinal complications. | ***Gastrointestinal complications***  **8 Cohort studies (N=1,436610)**  Treatment 2410/399,399 Control 2247/1037211  OR 2.29 (1.50-3.51)  Chi^2^ P<0.00001  **25 Case-control studies (N=74637)**  Treatment 3800/13610 Control 5512/61027  OR 3.81 (3.17-4.58)  Chi^2^ P<0.00001 | **Confounding factors by study design:**  NR but carries out subgroup analysis by type of drug  **Heterogeneity within study designs:**:  Significant heterogeneity: one set of cohort studies and one set of case-control studies  **Statistical analysis comparing study designs:** NR but authors state that there is a marked difference in pooled odds ratios. | **Cohort studies:** Significant increase P=0.0001  **Case-control studies:** Significant increase P<0.00001  **CI overlap:** Yes |
| Johnston et al 1998^13^ | Systematic review of oral contraceptives and subarachnoid hemorrahage | ***Subarachnoid hemorrahage***  **2 Cohort studies (person years=588,151)**  RR 1.92 (0.91-4.06)  Chi^2^ P=0.41  **10 Case-control studies (N=8,904)**  RR 1.40 (1.10-1.78)  Chi^2^ P=0.30 | **Confounding factors by study design:**  NR but presents risk ratio stratified by dose, smoking, hypertension, exposure classification and outcome measure.  **Heterogeneity within study designs:** No **s**ignificant heterogeneity: one set of cohort studies and one set of case-control studies (no significant heterogeneity when all studies are pooled)  **Statistical analysis comparing study designs:** Summary estimates from subgroups of studies were compared using a z statistic. The difference between risk ratio from cohort studies and case-control studies was not significant (p>0.10). | **Cohort studies:**  No significant difference  **Case-control studies:**  Significant increase  **CI overlap:** Yes |
| Koster et al 1995^14^ | Systematic review of oral contraceptives and venous thromboembolism. | ***Venous thromboembolism***  **6 Cohort studies (N=NR)**  RR 2.1 (0.3-16)  **8 Case-control studies (N=NR**)  RR 4.2 (1.3-14) | **Confounding factors by study design:**  NR but author states differences may be due to study bias  **Heterogeneity within study designs:** NR (Significant heterogeneity among all studies P<0.001)  **Statistical analysis comparing study designs:** NR | **Cohort studies:** No significant difference  **Case-control studies:**  Significant increase  **CI overlap:** Yes |
| Leipzig et al 1999^15^ | Systematic review of psychotrophic medications and falls. | ***Falls (psychotropics)***  **11 Cohort studies (N=NR)**  OR 1.66 (1.40-1.97)  **6 Case-control studies (N=NR)**  OR 2.57 (1.90-3.49)  **2 Cross sectional studies ((N=NR)**  OR 1.40 (1.08-1.81)  ***Falls (antidepressants)***  **11 Cohort studies (N=NR)**  OR 1.62 (1.23-2.14)  **12 Case-control studies (N=NR)**  OR 1.89 (1.41-2.52)  **4 Cross sectional studies (N=NR)**  OR 1.51 (1.16-1.98)  ***Falls (neuroleptics)***  **10 Cohort studies (N=NR)**  OR 1.90 (1.35-2.67)1  **10 Case-control studies (N=NR)**  OR 1.20 (0.90-1.61)  **2 Cross sectional studies (N=NR)**  OR 1.59 (1.18-2.13)  ***Falls (sedative/hypnotics)***  **9 Cohort studies (N=NR)**  OR 1.25 (0.98-1.60)  **9 Case-control studies (N=NR)**  OR 1.63 (1.31-2.02)  **4 Cross sectional studies (N=NR)**  OR 1.60 (1.41-1.82)  ***Falls (benzodiazepines)***  **8 Cohort studies (N=NR)**  OR 1.40 (1.11-1.76)  **3 Case-control studies (N=NR)**  OR 2.57 (1.46-4.51)  **2 Cross sectional studies (N=NR)**  OR 1.34 (0.95-1.88) | **Confounding factors by study design:** NR but stratification of studies by subject residence, community studies, age, ascertainment of medication and falls had no effect on the pooled odds ratios.  **Heterogeneity within study designs:** NR (Significant heterogeneity among all studies of psychotropics, neuroleptics, and seductive hypnotics but not the other interventions)  **Statistical analysis comparing study designs:** NR but authors state stratification by study design had no effect on the pooled odds ratios. No statistical analysis presented. | ***Psychotropics***  **Cohort studies:**  Significant increase  **Case-control studies:**  Significant increase  **Cross sectional studies:**  Significant increase  **CI overlap:** No, cohort studies have higher odds ratio than cross-sectional studies  ***Antidepressants***  **Cohort studies:** Significant increase  **Case-control studies:**  Significant increase  **Cross sectional studies:**  Significant increase  **CI overlap:** Yes  ***Neuroleptics***  **Cohort studies:** Significant increase  **Case-control studies:** No significant difference  **Cross sectional studies:**  significant increase  **CI overlap:** yes  ***Sedative/Hypnotics***  **Cohort studies:**  No significant difference  **Case-control studies:**  significant increase  **Cross sectional studies:**  significant increase  **CI overlap:** yes  ***Benzodiazepines***  **Cohort studies:**  significant increase  **Case-control studies:**  significant increase  **Cross sectional studies:**  No significant difference  **CI overlap:** yes |
| Leipzig et al 1999^16^ | Systematic review of cardiovascular medications and falls. | ***Falls (thiazides)***  **8 Cohort studies (N=NR)**  OR 1.05 (0.96-1.21)  **3 Case-control studies (N=NR)**  OR 1.97 (0.89-4.36)  **1 Cross sectional studies (N=NR)**  OR 1.15 (0.70-1.90)  ***Falls (loop diuretics)***  **7 Cohort studies (N=NR)**  OR 0.90 (0.68-1.18)  **3 Case-control studies (N=NR)**  OR 0.76 (0.51-1.16)  **1 Cross sectional study (N=NR)**  OR 1.49 (0.77-2.89)  ***Falls (digoxin)***  **9 Cohort studies (N=NR)**  OR 1.29 (1.01-1.65)  **5 Case-control studies (N=NR)**  OR 1.31 (0.91-1.87)  **3 Cross sectional studies (N=NR)**  OR 1.13 (0.90-1.42)  ***Falls (nitrates)***  **8 Cohort studies (N=NR)**  OR 1.29 (0.99-1.68)  **4 Case-control studies (N=NR)**  OR 0.87 (0.59-1.28)  **2 Cross sectional studies (N=NR)**  1.12 (0.82-1.54)  ***Falls (beta-blockers)***  **9 Cohort studies (N=NR)**  OR 1.00 (0.78-1.30)  **7 Case-control studies (N=NR)**  OR 0.83 (0.51-1.35)  **2 Cross sectional studies (N=NR)**  OR 0.87 (0.64-1.18)  ***Falls (calcium channel blockers)***  **8 Cohort studies (N=NR)**  OR 1.05 (0.82-1.36)  **4 Case-control studies (N=NR)**  OR 0.88 (0.54-1.43)  **1 Cross sectional study (N=NR)**  OR 0.69 (0.44-1.09)  ***Falls (ACE inhibitors)***  **7 Cohort studies (N=NR)**  OR 1.09 (0.76-1.55)  **2 Case-control studies (N=NR)**  OR 1.69 (0.89-3.21)  **1 Cross sectional study (N=NR)**  OR 1.19 (0.68-2.07)  ***Falls (Centrally acting antihypertensives)***  **4 Cohort studies (N=NR)**  OR 0.80 (0.39-1.66)  **5 Case-control studies (N=NR)**  OR 1.41 (0.71-2.79)  **2 Cross sectional studies (N=NR)**  OR 1.21 (0.85-1.73)  ***Falls (type 1A antiarrhythmics)***  **5 Cohort studies (N=NR)**  OR 0.95 (0.46-1.97)  **4 Case-control studies (N=NR)**  OR 3.68 (1.20-11.27)  **1 Cross sectional study (N=NR)**  OR 1.73 (0.87-3.41) | **Confounding factors by study design:**  NR but stratification of studies by subject residence, community studies, age, ascertainment of medication and falls had no effect on the pooled odds ratios.  **Heterogeneity within study designs:** NR (no significant heterogeneity with all studies)  **Statistical analysis comparing study designs:** NR but authors state stratification by study design had no effect on the pooled odds ratios. No statistical analysis presented. | ***Thiazides***  **Cohort studies:**  No significant difference  **Case-control studies:**  No significant difference  **Cross sectional studies:**  No significant difference  **CI overlap:** yes  ***Loop diuretics***  **Cohort studies:**  No significant difference  **Case-control studies:**  No significant difference  **Cross sectional studies:**  No significant difference  **CI overlap:** yes  ***Digoxin***  **Cohort studies:**  Significant increase  **Case-control studies:**  No significant difference  **Cross sectional studies:**  No significant difference  **CI overlap:** yes  ***Nitrates***  **Cohort studies:**  No significant difference  **Case-control studies:**  No significant difference  **Cross sectional studies:**  No significant difference  **CI overlap:** yes  ***Beta-blockers***  **Cohort studies:**  No significant difference  **Case-control studies:**  No significant difference  **Cross sectional studies:**  No significant difference  **CI overlap:** yes  ***Calcium channel blockers***  **Cohort studies:**  No significant difference  **Case-control studies:**  No significant difference  **Cross sectional studies:**  No significant difference  **CI overlap:** yes  **ACE inhibitors**  **Cohort studies:**  No significant difference  **Case-control studies:**  No significant difference  **Cross sectional studies:**  No significant difference  **CI overlap:** yes  ***Centrally acting antihypertensives***  **Cohort studies:**  No significant difference  **Case-control studies:**  No significant difference  **Cross sectional studies:**  No significant difference  **CI overlap:** yes  ***Type 1A antiarrhythmics***  **Cohort studies:**  No significant difference  **Case-control studies:**  Significant increase  **Cross sectional studies:**  No significant difference  **CI overlap:** yes |
| Loke et al 2008^17^ | Systematic review of thiazolidinediones and fractures | ***Fractures among women***  **1 Cohort study**  OR 1.38 (1.03-1.82)  **1 Case-control study**  OR 2.56 (1.43-4.58) | **Confounding factors by study design:**  NR but authors acknowledge that the trials contained relatively young participants and the case-control study involved an older population.  **Heterogeneity within study designs:** NR  **Statistical analysis comparing study designs:** NR | ***Fractures among women***  **Cohort study**  Significant increase  **Case-control study**  Significant increase  **CI overlap:** yes |
| MacLennan et al 1995^18^ | Systematic review of oestrogen replacement therapy and colorectal cancer . | ***Colorectal cancer***  **4 Cohort studies (N=169400)**  RR 0.91 (0.60-1.38)  Woolf’s test, P=0.89  **9 Case-control studies (N=8631)**  RR 0.92 (0.71-1.20)  Woolf’s test, P<0.01 | **Confounding factors by study design:**  Acknowledges insufficient information on dose duration to check variables.  **Heterogeneity within study designs:** No significant heterogeneity: one set of cohort studies.  Significant heterogeneity: one set of case-control studies.  **Statistical analysis comparing study designs:** NR | ***Colorectal cancer***  **Cohort studies:**  No significant difference  **Case-control studies:**  No significant difference  **CI overlap:** Yes |
| McGettigan and Henry 2006^19^ | Systematic review of NSAIDS and cardiovascular events. | ***Cardiovascular events (celecoxib)***  **3 Cohort studies (N=NR)**  RR 1.22 (0.69-2.16)  **8 Case-control studies (N=NR)**  RR 1.01 (0.90-1.13)  ***Cardiovascular events (rofecoxib < or = 25mg/d*)***  **2 Cohort studies (N=NR)**  RR 1.51 (0.73-3.13)  **3 Case-control studies (N=NR)**  RR 1.21 (1.08-1.36)  ***Cardiovascular events (rofecoxib > or = 25mg/d*)***  **2 Cohort studies (N=NR)**  RR 2.46 (1.29-4.71)  **4 Case-control studies (N=NR)**  RR 1.89 (1.43-2.51)  ***Cardiovascular events (naproxen)***  **3 Cohort studies (N=NR)**  RR 0.94 (0.85-1.04)  **12 Case-control studies (N=NR)**  RR 0.96 (0.84-1.10)  ***Cardiovascular events (diclofenac)***  **2 Cohort studies (N=NR)**  RR 1.36 (0.51-3.65)  **7 Case-control studies (N=NR)**  RR 1.36 (1.21-1.54)  ***Cardiovascular events (ibuprofen)***  **5 Cohort studies (N=NR)**  RR 1.12 (0.90-1.38)  **11 Case-control studies (N=NR)**  RR 1.06 (0.95-1.18)  ***Cardiovascular events (any/other NSAIDS)***  **5 Cohort studies (N=NR)**  RR 1.10 (0.95-1.29)  **14 Case-control studies (N=NR)**  RR 1.10 (0.98-1.24) | **Confounding factors by study design:**  NR  **Heterogeneity within study designs:** NR (Significant heterogeneity for all studies)  **Statistical analysis comparing study designs:** NR | ***Celecoxib***  **Cohort studies:**  No significant difference  **Case-control studies:**  No significant difference  **CI overlap:** yes  ***Rofecoxib < or = 25mg/d****  **Cohort studies:**  No significant difference  **Case-control studies:**  Significant increase  **CI overlap:** yes  ***Rofecoxib > or = 25mg/d****  **2 Cohort studies**  Significant increase  **4 Case-control studies**  Significant increase  **CI overlap:** yes  ***Naproxen***  **Cohort studies:**  No significant difference  **Case-control studies:**  No significant difference  **CI overlap:** yes  ***Diclofenac***  **Cohort studies:**  No significant difference  **Case-control studies:**  Significnat increase  **CI overlap:** yes  ***Ibuprofen***  **Cohort studies:**  No significant difference  **Case-control studies:**  No significant difference  **CI overlap:** yes  ***Any/Other NSAIDS***  **Cohort studies:**  No significant difference  **Case-control studies:**  No significant difference  **CI overlap:** yes |
| Ofman et al 2002^20^ | Systematic review of NSAIDs and severe upper gastrointestinal complications perforations, ulcers and bleeds. | ***Gastrointestinal complications perforations, ulcers and bleeds***  **9 Cohort studies (N=758776 patient-years)**  RR 2.7 (2.1-3.5)  **23 Case-control studies (N=25732)**  OR 3.0 (2.5-3.7) | **Confounding factors by study design:**  NR but states that data were insufficient to justify subgroup analysis by age, comorbid conditions, drug or dose.  **Heterogeneity within study designs:** NR (Only pooled homogeneous studies for each study design)  **Statistical analysis comparing study designs:** NR | **Cohort studies:** Significant increase  **Case-control studies:** Significant increase  **CI overlap:** Yes |
| Oger and Scarabin 1999^21^ | Systematic review of hormone replacement therapy and venous thromboembolism. | ***Venous thromboembolism***  **1 cohort study (N=NR)**  RR 2.1 (1.2-3.8)  **7 case-control studies (N=NR)**  RR 2.1 (1.4-3.0)  Chi^2^, P=NS | **Confounding factors by study design:**  NR  **Heterogeneity within study designs:** No significant heterogeneity: one set of case-control studies  **Statistical analysis comparing study designs:** NR | **Cohort study**  Significant increase  **Case-control studies**  Significant increase  **CI overlap:** yes |
| Salhab et al 2005^22^ | Systematic review of ovulation induction in IVF.and breast cancer. | ***Breast cancer***  **11 Cohort studies (N=NR)**  Treatment 601/60050 Control NR  RR 1.06 (0.94-1.19)****** (P=0.337)  **4 Case-control studies (N=22233)**  Cases 253/11303 Controls 273/10930  RR 0.88 (0.72-1.08)** (P=0.224) | **Confounding factors by study design:**  NR  **Heterogeneity within study designs:** NR  **Statistical analysis comparing study designs:** NR | **Cohort studies:** No significant difference  **Case-control studies:** No significant difference  **CI overlap:** Yes |
| Schwarz et al 2008^23^ | Systematic review of loratadine and hypospadias. | ***Hypospadias***  **2 Cohort studies**  OR 1.23 (0.32-4.69)  **2 Case-control studies**  OR 0.95 (0.43-2.08) | **Confounding factors by study design:** NR  **Heterogeneity within study designs:** NR  **Statistical analysis comparing study designs:** NR | **Cohort studies**  No significant difference  **Case-control studies**  No significant difference  **CI overlap:** yes |
| Scott et al 2007^24^ | Systematic review of NSAIDs and myocaridial infarction***.*** | ***Myocaridial infarction (naproxen)***  **4 Cohort studies (N=571679 patient years)**  RR 0.96 (0.90-1.03)  **11 Case-control studies (N=384324)**  OR 1.03 (0.83-1.29)  ***Myocaridial infarction (ibuprofen)***  **3 Cohort studies (N=552150** **patient years)**  RR 0.90 (0.82-0.97)  **8 Case-control studies (N=286089)**  OR 1.08 (0.80-1.46)  ***Myocaridial infarction (celecoxib)***  **3 Cohort studies (N=330651** **patient years)**  RR 1.06 (1.00-1.13)  **7 Case-control studies (N=319841)**  OR 1.01 (0.73-1.39)  ***Myocaridial infarction (rofecoxib)***  **3 Cohort studies (N=322443** **patient years)**  RR 1.25 (1.17-1.34)  **7 Case-control studies (N=203487)**  OR 1.19 (0.70-2.01) | **Confounding factors by study design:** Acknowledges that discrepancies may arise from selection of controls and populations studied.  **Heterogeneity within study designs:** NR (Significant heterogeneity among all 6 cohort studies for a*ll NSAIDS*  Chi^2^ P<0.001, I^2^=92.1% and for all 14 case-control studies Chi^2^ P<0.001, I^2^=97.9%)  **Statistical analysis comparing study designs:** NR | ***Naproxen***  **Cohort studies:** No significant difference  **Case-control studies:** No significant difference  **CI overlap:** yes  ***Ibuprofen***  **Cohort studies:**  Significant decrease  **Case-control studies:**  No significant difference  **CI overlap:** yes  **Celecoxib**  **Cohort studies:** No significant difference  **Case-control studies:** No significant difference  **CI overlap:** yes  **Rofecoxib**  **Cohort studies:** Significant increase  **Case-control studies:** No significant difference  **CI overlap:** yes |
| Scott et al 2008^25^ | Systematic review of NSAIDS and cardiac failure. | ***Cardiac failure***  **2 Cohort studies (82785 patient years)**  RR 1.97 (1.73-2.25)  Chi^2^ P=0.33, I^2^= 0%  **5 Case-control studies (N=50519)**  OR 1.36 (0.99-1.85)  Chi^2^ P<0.001, I^2^= 90.9% | **Confounding factors by study design:** NR but discusses the problems of over the counter NSAIDS in observational studies and that one case-control study excluded patients with previous cardiac failure.  **Heterogeneity within study designs:** No significant heterogeneity: one set of cohort studies.  Significant heterogeneity: one set of case-control studies.  **Statistical analysis comparing study designs:** NR | **Cohort studies:** Significant increase  **Case-control studies:** No significant difference  **CI overlap:** yes |
| Smith et al 2003^26^ | Systematic review of hormonal contraceptives and cervical cancer. | ***Cervical cancer (short duration users of contraceptives)***  **4 Cohort studies (N=NR)**  RR 1.8 (1.4-2.4)  Chi^2^ P>0.1  **16 Case-control studies (N=NR)**  RR 1.1 (1.0-1.2)  Chi^2^ P=0.004  ***Cervical cancer (medium duration users of contraceptives)***  **4 Cohort studies (N=NR)**  RR 2.2 (1.7-2.9)  Chi^2^ P=0.007  **17 Case-control studies (N=NR)**  RR 1.5 (1.4-1.7)  Chi^2^ P=0.03  ***Cervical cancer (long duration users of contraceptives)***  **3 Cohort studies (N=NR)**  RR 3.3 (2.4-4.5)  Chi^2^ P=0.02  **10 Case-control studies (N=NR)**  RR 2.0 (1.8-2.3)  Chi^2^ P=0.03 | **Confounding factors by study design:** NR but conducts subgroup analysis on other factors such as HPV status, sexual partners, cervical screening, smoking, barrier contraceptives, country, invasive cervical cancer, in situ cervical cancer, squamous cervical cancer, adrenocarcinoma of the cervix but not by study design.  **Heterogeneity within study designs: N** No significant heterogeneity: one set of cohort studies  Significant heterogeneity: 2 sets of cohort studies and 3 sets of case-control studies (significant heterogeneity when all studies are pooled)  **Statistical analysis comparing study designs:** NR but authors state that RR consisitently higher in the cohort studies than case-control studies even within stratified categories of duration. States that the reason for this is unclear. | ***Short duration users***  **Cohort studies:**  Significant increase  **Case-control studies:**  No significant difference  **CI overlap:** no  ***Medium duration users***  **Cohort studies:**  Significant increase  **Case-control studies:**  Significant increase  **CI overlap:** yes  ***Long duration users***  **Cohort studies:**  Significant increase  **Case-control studies:**  Significant increase  **CI overlap:** no |
| Takkouche et al 2007^27^ | Systematic review of psychotropic medications and fracture. | ***Fracture (benzodiazepines)***  **7 Cohort studies (N=NR)**  RR 1.31 (1.18-1.45)  Q test P=0.36  **16 Case-control studies (N=NR)**  RR 1.36 (1.23-1.51)  Q test P=0.0001  ***Fracture (antidepressants)***  **3 Cohort studies (N=NR)**  RR 1.28 (1.04-1.58)  Q test P=0.79  **13 Case-control studies (N=NR)**  RR 1.66 (1.41-1.96)  Q test P=0.00001  ***Fracture (non-barbiturate antiepileptic drugs)***  **4 Cohort studies (N=NR)**  RR 1.34 (0.96-1.88)  Q test P=0.21  **9 Case-control studies**  RR 1.64 (1.24-2.16)  Q test P=0.00001  ***Fracture (antipsychotics)***  **2 Cohort studies**  RR 1.11 (0.70-1.75)  Q test P=0.42  **10 Case-control studies**  RR 1.68 (1.32-2.14)  Q test P=0.00001  ***Fracture (hypnotics)***  **3 Cohort studies**  RR 1.04 (0.86-1.25)  Q test P=0.49  **10 Case-control studies**  RR 1.22 (0.97-1.54)  Q test P=0.008  ***Fracture (opiods)***  **3 Cohort studies**  RR 1.32 (1.02-1.70)  Q test P=0.18  **3 Case-control studies**  RR 1.42 (1.04-1.93)  Q test P=0.001 | **Confounding factors by study design:** NR but conducts subgroup analysis by selected characteristics. ***Benzodiazepines:*** Did not find any evidence of a substantial difference in pooled RRs according to duration of action, study quality score or by limiting to hip fractures alone. ***Antidepressants:*** Did not find any evidence of a substantial difference in pooled RRs according to study quality score. ***Antiepileptic drugs:*** Low quality studies had higher RR. ***Antipsychotics:*** Results similar according to anatomic site of fracture and quality scoring. ***Hypnotics:*** Results similar according to quality scoring.  **Heterogeneity within study designs:** No significant heterogeneity: 6 sets of cohort studies.  Significant heterogeneity: 6 sets of case-control studies.  **Statistical analysis comparing study designs:** NR but authors state that they did not find any substantial difference in pooled risk ratio according to study design for studies of Benzodiazepines and that cohort studies showed a lower pooled risk ratio than case-control studies for studies of antidepressants. | ***Benzodiazepines***  **Cohort studies:**  Significant increase  **Case-control studies:**  Significant increase  **CI overlap:** yes  ***Antidepressants***  **Cohort studies:**  Significant increase  **Case-control studies:**  Significant increase  **CI overlap:** yes  ***Antiepileptic* drugs**  **Cohort studies:**  No significant difference  **Case-control studies:** Significant increase  **CI overlap:** yes  ***Antipsychotics***  **Cohort studies:** No significant difference  **Case-control studies:** Significant increase  **CI overlap:** yes  ***Hypnotics***  **Cohort studies:** No significant difference  **Case-control studies:** No significant difference  **CI overlap:** yes  ***Opiods***  **Cohort studies:** Significant increase  **Case-control studies:** Significant increase  **CI overlap:** yes |
| Torloni et al 2009^28^ | Systematic review of ultrasonography in pregnancy | ***Low birth weight***  **6 Cohort studies (N=18622)**  OR 1.11 (0.84-1.46)  I^2^=72.8%  **1 Case control study(N=12,546)**  1.38 (1.25-1.51) | **Confounding factors by study design:** NR  **Heterogeneity within study designs:**  Significant heterogeneity: one set of cohort studies.  **Statistical analysis comparing study designs:** NR | ***Low birth weight***  **Cohort studies:** No significant difference  **Case control study:** Significant increase  **CI overlap:** yes |
| Woolcott et al 2009^29^ | Systematic review of falls in the elderly | ***Falls (antihypertensives)***  **3 cohort studies (N=NR)**  OR 1.34 (0.93-1.91)  **2 case-control studies (N=NR)**  OR 1.09 (0.80-1.50)  **1 cross-sectional study (N=NR)**  OR 1.11 (0.78-1.58)  ***Falls (diuretics)***  **1 cohort study (N=NR)**  OR 1.05 (0.97-1.15)  **5 case-control studies (N=NR)**  OR 1.11 (0.94-1.32)  **3 cross-sectional studies (N=NR)**  OR 1.11 (1.00-1.24)  ***Falls (b-blockers)***  **1 case-control study (N=NR)**  OR 0.87 (0.55-1.37)  **3 cross-sectional studies (N=NR)**  OR 1.02 (0.79-1.24)  ***Falls (sedatives/hypnotics)***  **3 cohort studies (N=NR)**  OR 1.24 (1.05-1.45)  **1 case-control study (N=NR)**  OR 1.62 (1.31-2.00)  **3 cross-sectional studies (N=NR)**  OR 1.56 (1.39-1.76) | **Confounding factors by study design:** NR  **Heterogeneity within study designs:** NR  **Statistical analysis comparing study designs:** NR | ***Antihypertensives***  **Cohort studies:** No significant difference  **Case-control studies:** No significant difference  **Cross-sectional study:** No significant difference  **CI overlap:** yes  ***Diuretics***  **Cohort study:** No significant difference  **Case-control studies:** No significant difference  **Cross-sectional studies:** No significant difference  **CI overlap:** yes  ***B-Blockers***  **Case-control study:** No significant difference  **Cross-sectional studies:** No significant difference  **CI overlap:** yes  ***Sedatives/hypnotics***  **Cohort studies:** Significant increase  **Case-control study:** Significant increase  **Cross-sectional studies:** Significant increase  **CI overlap:** yes |

**Key**

RR – Risk ratio

N – Number of study participants

WMD – Weighted Means Difference

OR – Odds Ratio

NR – Not reported

CI – Confidence Interval

**- data were calculated from information presented in paper

**References**

1. Agency for Healthcare Research and Quality. *Hormone Replacement Therapy and Risk of Venous Thromboembolism*. Rockville, MD: Agency for Healthcare Research and Quality 2002.

2. Bager P, Whohlfahrt J, Westergaard T. Caesarean delivery and risk of atopy and allergic disease: meta-analysis. *Clin Exp Allergy* 2008;38:634-42.

3. Bergendal A, Odlind V, Persson I, Kieler H. Limited knowledge on progestogen-only contraception and risk of venous thromboembolism. *Acta Obstet Gynecol Scand* 2009;88:261-66.

4. Bollini P, Garcia RLA, Pérez GS, Walker AM. The impact of research quality and study design on epidemiologic estimates of the effect of nonsteroidal anti-inflammatory drugs on upper gastrointestinal tract disease. *Arch Intern Med.* 1992;152:1289-95.

5. Capurso G, Schünemann HJ, Terrenato I, Moretti A, Koch M, Muti P, et al. Meta-analysis: the use of non-steroidal anti-inflammatory drugs and pancreatic cancer risk for different exposure categories. *Aliment Pharmacol Ther* 2007;26:1089-99.

6. Chan WS, Ray J, Wai EK, Ginsburg S, Hannah ME, Corey PN, et al. Risk of stroke in women exposed to low-dose oral contraceptives: a critical evaluation of the evidence. *Arch Intern Med* 2004;164:741-7.

7. Dolovich LR, Addis A, Vaillancourt JM, Power JD, Koren G, Einarson TR. Benzodiazepine use in pregnancy and major malformations or oral cleft: meta-analysis of cohort and case-control studies. *BMJ* 1998;317:839-43.

8. Douketis JD, Ginsberg JS, Holbrook A, Crowther M, Duku EK, Burrows RF. A reevaluation of the risk for venous thromboembolism with the use of oral contraceptives and hormone replacement therapy. *Arch Intern Med.* 1997;157:1522-30.

9. Garg PP, Kerlikowske K, Subak L, Grady D. Hormone replacement therapy and the risk of epithelial ovarian carcinoma: a meta-analysis. *Obstet Gynecol* 1998;92:472-9.

10. Gillum LA, Mamidipudi SK, Johnston SC. Ischemic stroke risk with oral contraceptives: a meta-analysis. *JAMA* 2000;284:72-8.

11. Grady D, Gebretsadik T, Kerlikowske K, Ernster V, Petitti D. Hormone replacement therapy and endometial cancer risk: a meta-analysis. *Obstet Gynecol* 1995;85:304-13.

12. Henry D, McGettigan P. Epidemiology overview of gastrointestinal and renal toxicity of NSAIDs. *Int J Clin Pract Suppl* 2003;135:43-9.

13. Johnston SC, Colford JM, Jr., Gress DR. Oral contraceptives and the risk of subarachnoid hemorrhage. *Neurology* 1998;51:411-8.

14. Koster T, Small RA, Rosendaal FR, Helmerhorst FM. Oral contraceptives and venous thromboembolism: a quantitative discussion of the uncertainties. *J Intern Med* 1995;238:31-7.

15. Leipzig RM, Cumming RG, Tinetti ME. Drugs and falls in older people: a systematic review and meta-analysis: I. Psychotropic drugs. *J Am Geriatr Soc* 1999;47:30-9.

16. Leipzig RM, Cumming RG, Tinetti ME. Drugs and falls in older people: a systematic review and meta-analysis: II. Cardiac and analgesic drugs. *J Am Geriatr Soc* 1999;47:40-50.

17. Loke YK, Singh S, Furberg CD. Long-term use of thiazolidinediones and fractures in type 2 diabetes: A meta-analysis. *CMAJ* 2008;180:32-9.

18. MacLennan SC, MacLennan AH, Ryan P. Colorectal cancer and oestrogen replacement therapy. A meta-analysis of epidemiological studies. *Med J Aust* 1995;162:491-3.

19. McGettigan P, Henry D. Cardiovascular risk and inhibition of cyclooxygenase: a systematic review of the observational studies of selective and nonselective inhibitors of cyclooxygenase 2. *JAMA* 2006;296:1633-44.

20. Ofman JJ, MacLean CH, Straus WL, Morton SC, Berger ML, Roth EA, et al. A meta-analysis of severe upper gastrointestinal complications of non-steroidal anti-inflammatory drugs. *J Rheumatol* 2002;29:804-12.

21. Oger E, Scarabin PY. Assessment of the risk for venous thromboembolism among users of hormone replacement therapy. *Drugs Aging* 1999;14:55-61.

22. Salhab M, Al Sarakbi W, Mokbel K. In vitro fertilization and breast cancer risk: a review. *Int J Fertil Womens Med* 2005;50:259-66.

23. Schwarz EB, Moretti ME, Nayak S, Koren G. Risk of hypospadias in offspring of women using loratadine during pregnancy: a systematic review and meta-analysis. *Drug Saf* 2008;31:775-88.

24. Scott PA, Kingsley GH, Smith CM, Choy EH, Scott DL. Non-steroidal anti-inflammatory drugs and myocardial infarctions: comparative systematic review of evidence from observational studies and randomised controlled trials. *Ann Rheum Dis* 2007;66:1296-304.

25. Scott PA, Kingsley GH, Scott DL. Non-steroidal anti-inflammatory drugs and cardiac failure: meta-analysis of observational studies and randomised controlled trials. *Eur J Heart Fail* 2008;10:1102-7.

26. Smith JS, Green J, Berrington de Gonzalez A, Appleby P, Peto J, Plummer M, et al. Cervical cancer and use of hormonal contraceptives: a systematic review. *Lancet* 2003;36:1159-67.

27. Takkouche B, Montes-Martínez A, Gill SS, Etminan M. Psychotropic medications and the risk of fracture: a meta-analysis. *Drug Saf* 2007;30:171-84.

28. Torloni MR, Vedmedovska N, Merialdi M, Betran AP, Allen T, Gonzales R, et al. Safety of ultrasonography in pregnancy: WHO systematic review of the literature and meta-analysis. *Ultrasound Obstet Gynecol* 2009;33:599-608.

29. Woolcott JC, Richardson KJ, Wiens MO, Patel B, Marin J, Khan KM, et al. Meta-analysis of the impact of 9 medication classes on falls in elderly persons. *Arch Intern Med* 2009;169:1952-60.
