# Supplementary material for: Comparison of Pooled Risk Estimates for Adverse Effects from Different Observational Study Designs: Methodological Overview
Source: PLoS One. 2013 Aug 20;8(8):e71813. doi: 10.1371/journal.pone.0071813 (PMC3748094; doi:10.1371/journal.pone.0071813)
Supplement: Appendix S3 — Excluded Studies and Reasons for Exclusion. (DOCX) [file pone.0071813.s003.docx]

Appendix 3: Excluded Studies

67 studies were excluded from this systematic review. Nearly a third of these studies (20/68) did not compare a formally recognised study design (such as RCTs, cohort studies or case-control studies).^1-20^ Thirteen studies were excluded because their hypothesis stated that the intervention had a protective (beneficial) effect.^21-32^ 23 studies did not present the pooled data for each study design ^33-55^ 5 studies did not compare similar data, for example, similar adverse effects,^56, 57^ similar comparators,^58^ or similar outcome measures.^59, 60^ For three studies only the abstract was available. ^61-63^ Two studies contained duplicate data from studies already included,^64, 65^ one study did not include a health care intervention ^66^ and lastly one study did not measure the magnitude or direction of any adverse effects but looked at the decline in risk.^67^

**References**

1. Blankensteijn JD. Mortality and morbidity rates after conventional abdominal aortic aneurysm repair. *Semin Interv Cardiol* 2000;5:7-13.

2. Choi PT, Galinski SE, Takeuchi L, Lucas S, Tamayo C, Jadad AR. PDPH is a common complication of neuraxial blockade in parturients: a meta-analysis of obstetrical studies. *Can J Anaesth* 2003;50:460-9.

3. Collaborative Group on Hormonal Factors in Breast Cancer. Breast cancer and hormonal contraceptives: collaborative reanalysis of individual data on 53 297 women with breast cancer and 100 239 women without breast cancer from 54 epidemiological studies. *Lancet* 1996;347:1713-27.

4. Curran TJ, Borzotta AP. Complications of primary repair of colon injury: literature review of 2,964 cases. *Am J Surg* 1999;177:42-7.

5. Dezfulian C, Lavelle J, Nallamothu BK, Kaufman SR, Saint S. Rates of infection for single-lumen versus multilumen central venous catheters: a meta-analysis. *Crit Care Med* 2003;31:2385-90.

6. Eikelboom JW, Mehta SR, Pogue J, Yusuf S. Safety outcomes in meta-analyses of phase 2 vs phase 3 randomized trials: Intracranial hemorrhage in trials of bolus thrombolytic therapy. *JAMA* 2001;285:444-50.

7. García Rodríguez LA, Hernández-Díaz S, de Abajo FJ. Association between aspirin and upper gastrointestinal complications: systematic review of epidemiologic studies. *Br J Clin Pharmacol* 2001;52:563-71.

8. Gordon CE, Feller-Kopman D, Balk EM, Smetana GW. Pneumothorax following thoracentesis: a systematic review and meta-analysis. *Arch Intern Med* 2010;170:332-39.

9. Hebert C, Delaney JA, Hemmelgarn B, Lévesque LE, Suissa S. Benzodiazepines and elderly drivers: a comparison of pharmacoepidemiological study designs. *Pharmacoepidemiol Drug Saf* 2007;16:845-9.

10. Levine MA, Hamet P, Novosel S, Jolain B. A prospective comparison of four study designs used in assessing safety and effectiveness of drug therapy in hypertension management. *Am J Hypertens* 1997;10:1191-200.

11. Marang-van de Mheen PJ, Hollander EJF, Kievit J. Effects of study methodology on adverse outcome occurrence and mortality. *International Journal for Quality in Health Care* 2007;19:399-406.

12. Marra F, Lynd L, Coombes M, Richardson K, Legal M, Fitzgerald JM, et al. Does antibiotic exposure during infancy lead to development of asthma?: a systematic review and metaanalysis. *Chest* 2006;129:610-8.

13. Martel N, Lee J, Wells PS. Risk for heparin-induced thrombocytopenia with unfractionated and low-molecular-weight heparin thromboprophylaxis: a meta-analysis. *Blood* 2005;106:2710-5.

14. Molloy D, Kaloo PD, Cooper M, Nguyen TV. Laparoscopic entry: a literature review and analysis of techniques and complications of primary port entry. *Aust N Z J Obstet Gynaecol* 2002;42:246-54.

15. Rothwell PM, Slattery J, Warlow CP. A systematic review of the risks of stroke and death due to endarterectomy for symptomatic carotid stenosis. *Stroke* 1996;27:260-65.

16. Safdar N, Said A, Gangnon RE, Maki DG. Risk of hemolytic uremic syndrome after antibiotic treatment of escherichia coli O157:H7 enteritis. *JAMA* 2002;288:996-1001.

17. Vamvakas EC. Perioperative blood transfusion and cancer recurrence: meta-analysis for explanation. *Transfusion* 1995;35:760-8.

18. Viboud C, Boelle, P.Y., Kelly, J., Auquier, A., Schlingmann, J., Roujeau, J.C., Flahault, A. Comparison of the statistical efficiency of case-crossover and case-control designs: Application to sever cutaneous adverse reactions. *J Clin Epidemiol* 2001;54:1218-27.

19. Glanz JM, McClure DL, Xu S, Hambidge SJ, Lee M, Kolczak MS, et al. Four different study designs to evaluate vaccine safety were equally validated with contrasting limitations. *J Clin Epidemiol* 2006;59:808-18.

20. van Staa TP, Smeeth L, Persson I, Parkinson J, Leufkens HG. Evaluating drug toxicity signals: is a hierarchical classification of evidence useful or a hindrance? *Pharmacoepidemiol Drug Saf* 2008;17:475-84.

21. Beral V, Doll R, Hermon C, Peto R, Reeves G. Ovarian cancer and oral contraceptives: collaborative reanalysis of data from 45 epidemiological studies including 23,257 women with ovarian cancer and 87,303 controls. *Lancet* 2008;371:303-14.

22. Bonovas S, Filioussi K, Flordellis CS, Sitaras NM. Statins and the risk of colorectal cancer: a meta-analysis of 18 studies involving more than 1.5 million patients. *J Clin Oncol* 2007;25:3462-8.

23. Etminan M, Gill S, Samii A. Effect of non-steroidal anti-inflammatory drugs on risk of Alzheimer's disease: systematic review and meta-analysis of observational studies. *BMJ* 2003;327:128.

24. Fernandez E, La Vecchia C, Balducci A, Chatenoud L, Franceschi S, Negri E. Oral contraceptives and colorectal cancer risk: a meta-analysis. *British Journal of Cancer* 2001;84:722-7.

25. Hébert-Croteau N. A meta-analysis of hormone replacement therapy and colon cancer in women. *Cancer Epidemiol Biomarkers Prev* 1998;7:653-9.

26. Kashyap S, Moher D, Fung MF, Rosenwaks Z. Assisted reproductive technology and the incidence of ovarian cancer: a meta-analysis. *Obstet Gynecol* 2004;103:785-94.

27. Larsson SC, Giovannucci E, Bergkvist L, Wolk A. Aspirin and nonsteroidal anti-inflammatory drug use and risk of pancreatic cancer: a meta-analysis. *Cancer Epidemiol Biomarkers Prev* 2006;15:2561-4.

28. Pladevall-Vila M, Delclos GL, Varas C, Guyer H, Brugues TJ, Anglada AA. Controversy of oral contraceptives and risk of rheumatoid arthritis: meta-analysis of conflicting studies and review of conflicting meta-analyses with special emphasis on analysis of heterogeneity. *American Journal of Epidemiology* 1996;144:1-14.

29. Reynolds F, Sharma SK, Seed PT. Analgesia in labour and fetal acid-base balance: a meta-analysis comparing epidural with systemic opioid analgesia. *BJOG* 2002;109:1344-53.

30. Toh S, Hernández-Díaz S. Statins and fracture risk. A systematic review. *Pharmacoepidemiol Drug Saf* 2007;16:627-40.

31. Wiens M, Etminan M, Gill SS, Takkouche B. Effects of antihypertensive drug treatments on fracture outcomes: a meta-analysis of observational studies. *J Intern Med* 2006;260:350-62.

32. Yaffe K, Sawaya G, Lieberburg I, Grady D. Estrogen therapy in postmenopausal women: effects on cognitive function and dementia. *JAMA* 1998;279:688-95.

33. Brumback BA, Holmes LB, Ryan LM. Adverse effects of chorionic villus sampling: a meta-analysis. *Stat Med* 1999;18:2163-75.

34. Col NF, Pauker SG. The discrepancy between observational studies and randomized trials of menopausal hormone therapy: did expectations shape experience? *Annals of Internal Medicine* 2003;139:923-29.

35. Costa HL, Doyle P. Influence of oral contraceptives in the development of post-molar trophoblastic neoplasia--a systematic review. *Gynecol Oncol* 2006;100:579-85.

36. Greiser CM, Greiser EM, Dören M. Menopausal hormone therapy and risk of breast cancer: a meta-analysis of epidemiological studies and randomized controlled trials. *Hum Reprod Update* 2005;11:561-73.

37. Grullon KE, Grimes DA. The safety of early postpartum discharge: a review and critique. *Obstet Gynecol* 1997;90:860-5.

38. Hall WD, Lucke J. How have the selective serotonin reuptake inhibitor antidepressants affected suicide mortality? . *Aust N Z J Psychiatry* 2006;40:941-50.

39. Hawkey CJ. Non-steriodal anti-inflammatory drugs and peptic ulcers: facts and figures multiply, but do they add up? *BMJ* 1990;300:278-84.

40. Janowsky EC, Kupper LL, Hulka BS. Meta-analysis of the relation between silicone breast implants and the risk of connective-tissue disease. *N Engl J Med* 2000;342:781-90.

41. Katerndahl DA, Realini JP, Cohen PA. Oral contraceptive use and cardiovascular disease: is the relationship real or due to study bias? . *J Fam Pract* 1992;35:147-57.

42. Kuoppala J, Lamminpaa A, Pukkala E. Statins and cancer: a systematic review and meta-analysis. *European Journal of Cancer* 2008;44:2122-32.

43. Lawlor DA, Jüni P, Ebrahim S, Egger M. Systematic review of the epidemiologic and trial evidence of an association between antidepressant medication and breast cancer. *J Clin Epidemiol* 2003;56:155-63.

44. Loke YK, Jeevanantham V, Singh S. Bisphosphonates and atrial fibrillation: systematic review and meta-analysis. *Drug Saf* 2009;32:219-28.

45. Magee LA, Bull SB, Koren G, Logan A. The generalizability of trial data; a comparison of beta-blocker trial participants with a prospective cohort of women taking beta-blockers in pregnancy. *European Journal of Obstet Gynecol and Reproductive Biology* 2001;94:205-10.

46. Martin G. Conflicting clinical trial data: a lesson from albumin. *Critical care* 2005;9:649-50.

47. Miwa LJ, Jones JK, Pathiyal A, Hatoum H. Value of epidemiologic studies in determining the true incidence of adverse events. The nonsteroidal anti-inflammatory drug story. *Arch Intern Med.* 1997;157:2129-36.

48. Nakhai-Pour HR, Berard A. Major malformations after first-trimester exposure to aspirin and NSAIDS. *Expert Review of Clinical Pharmacology* 2008;1:605-16.

49. Padwal R, Laupacis A. Antihypertensive therapy and incidence of type 2 diabetes: a systematic review. *Diabetes Care* 2004;27:247-55.

50. Rossi AC, Knapp DE, Anello C, O'Neill RT, Graham CF, Mendelis PS, et al. Discovery of adverse drug reactions. A comparison of selected phase IV studies with spontaneous reporting methods. *JAMA* 1983;249:2226-28.

51. Shah NR, Borenstein J, Dubois RW. Postmenopausal hormone therapy and breast cancer: a systematic review and meta-analysis. *Menopause* 2005;12:668-78.

52. Steffensmeier JJ, Ernst ME, Kelly M, Hartz AJ. Do randomized controlled trials always trump case reports? A second look at propranolol and depression. *Pharmacotherapy* 2006;26:162-67.

53. Steinberg KK, Smith SJ, Thacker SB, Stroup DF. Breast cancer risk and duration of estrogen use: the role of study design in meta-analysis. *Epidemiology* 1994;5:415-21.

54. Thavagnanam S, Fleming J, Bromley A, Shields MD, Cardwell CR. A meta-analysis of the association between Caesarean section and childhood asthma. *Clinical & Experimental Allergy* 2007;38:629-33.

55. Uboweja A, Malhotra S, Pandhi P. Effect of inhaled corticosteroids on risk of development of cataract: a meta-analysis. *Fundam Clin Pharmacol* 2006;20:305-9.

56. Demicheli V, Rivetti A, Di Pietrantonj C, Jefferson T. *Systematic review of adverse events following HBV immunization*. Geneva, Switzerland: World Health Organization; 2003.

57. Ernst E, Rand JI, Barnes J, Stevinson C. Adverse effects profile of the herbal antidepressant St. John's wort (Hyperisum perforatum L.). *Eur J Clin Pharmacol* 1998;54:589-94.

58. Egger M, Schneider M, Davey Smith G. Spurious precision? Meta-analysis of observational studies. *BMJ* 1998;316.

59. O'Brien L, Einarson TR, Sarkar M, Einarson A, Koren G. Does paroxetine cause cardiac malformations? *Journal of Obstetrics and Gynaecology Canada* 2008;30:696-701.

60. Singh S, Loke YK. Statins and pancreatitis: a systematic review of observational studies and spontaneous case reports. *Drug Saf* 2006;29:1123-32.

61. Henry D, Moxey A, O'Connell D. Agreement between randomized and non-randomized studies - the effects of bias and confounding. In: *9th Annual Cochrane Colloquium Abstracts*; 2001 October; 9th Annual Cochrane Colloquium Abstracts: Lyon. 2001.

62. Vandermeer B, Wiebe N, Buscemi N, Hartling L, Klassen T. Adverse events and adverse effects in systematic reviews: some methodological issues [abstract]. *12th Cochrane Colloquium: Bridging the Gaps; 2004 Oct 2-6; Ottawa, Ontario, Canada* 2004:199.

63. Zhang WY. Evidence from case-control and cohort studies for adverse drug reaction - a case study of analgesic nephropathy. *9th Annual Cochrane Colloquium Abstracts, October 2001 in Lyon* 2001.

64. Miller J, Chan BKS, Nelson HD. Postmenopausal estrogen replacement and risk for venous thromboembolism: A systematic review and meta-analysis for the US Preventive Services Task Force. *Annals of Internal Medicine* 2002;136:680-90. Available from: ISI:000175357900006

65. Meenan RT, Saha S, Chou R, Swarztrauber K, Krages KP, et al. *AHRQ Evidence report:: Effectiveness and Cost-Effectiveness of Echocardiography and Carotid Imaging in the Management of Stroke*; 2002.

66. Boyd NF, Stone J, Vogt KN, Connelly BS, Martin LJ, Minkin S. Dietary fat and breast cancer risk revisited: a meta-analysis of the published literature. *Br J Cancer* 2003;89:1672-85.

67. Schaffer D, Florin T, Eagle C, Marschner I, Singh G, Grobler M, et al. Risk of serious NSAID-related gastrointestinal events during long-term exposure: a systematic review. *Med J Aust* 2006;185:501-6.
