# Supplementary material for: Comparison of Pooled Risk Estimates for Adverse Effects from Different Observational Study Designs: Methodological Overview
Source: PLoS One. 2013 Aug 20;8(8):e71813. doi: 10.1371/journal.pone.0071813 (PMC3748094; doi:10.1371/journal.pone.0071813)
Supplement: Appendix S4 — Selection of outcomes and handling of duplicate data. (DOCX) [file pone.0071813.s004.docx]

**Appendix 4: Duplicate data decisions**

A major problem with the methodological evaluations was the issue of double data. Using the same data set more than once or multiple related outcomes in any analysis may make the data appear inappropriately homogenous and may present relationships which do not exist. The following decisions were, therefore, implemented before data entry into STATA.

1. Where both random effects models and fixed effects models were presented^1^ the random effects models were selected over the fixed effects models as they represent a more conservative approach, and studies were often heterogeneous (where measured).
2. Primary outcomes of fractures,^2^ and low birth weight,^3^ were selected in preference to the secondary outcomes of bone mineral density^2^ and preterm birth and other perinatal outcomes.^3^

**References**

1. Agency for Healthcare Research and Quality. *Hormone Replacement Therapy and Risk of Venous Thromboembolism*. Rockville, MD: Agency for Healthcare Research and Quality 2002.

2. Loke YK, Singh S, Furberg CD. Long-term use of thiazolidinediones and fractures in type 2 diabetes: A meta-analysis. *CMAJ* 2008;180:32-9.

3. Torloni MR, Vedmedovska N, Merialdi M, Betran AP, Allen T, Gonzales R, et al. Safety of ultrasonography in pregnancy: WHO systematic review of the literature and meta-analysis. *Ultrasound Obstet Gynecol* 2009;33:599-608.
