# Supplementary material for: Comparison of Pooled Risk Estimates for Adverse Effects from Different Observational Study Designs: Methodological Overview
Source: PLoS One. 2013 Aug 20;8(8):e71813. doi: 10.1371/journal.pone.0071813 (PMC3748094; doi:10.1371/journal.pone.0071813)
Supplement: Appendix S5 — Forest Plot: meta-analysis of RORs from cohort/cross-sectional studies versus case-control studies. (DOCX) [file pone.0071813.s005.docx]

**Appendix 5: Meta-analysis of RORs from cohort/ cross-sectional studies versus case-control studies**
