# Supplementary material for: Comparison of Pooled Risk Estimates for Adverse Effects from Different Observational Study Designs: Methodological Overview
Source: PLoS One. 2013 Aug 20;8(8):e71813. doi: 10.1371/journal.pone.0071813 (PMC3748094; doi:10.1371/journal.pone.0071813)
Supplement: Appendix S6 — Funnel plot of distribution of RORs from (i) meta-analyses of cohort studies compared to case-control studies (ii) meta-analyses of cross-sectional studies compared to case-control studies. (DOCX) [file pone.0071813.s006.docx]

**Appendix 6**

Figure: Funnel Plot of distribution of discrepancy (ln ROR) between (i) cohorts or (ii) cross-sectional studies as compared to case control studies:
