# Supplementary material for: Comparison of Pooled Risk Estimates for Adverse Effects from Different Observational Study Designs: Methodological Overview
Source: PLoS One. 2013 Aug 20;8(8):e71813. doi: 10.1371/journal.pone.0071813 (PMC3748094; doi:10.1371/journal.pone.0071813)
Supplement: Appendix S7 — Sensitivity analysis based on exclusion of meta-analyses covering the same adverse effect where there is some overlap in the included primary studies. (DOCX) [file pone.0071813.s007.docx]

**Appendix 7**

*Sensitivity analysis*

There was some, but not complete overlap of primary studies in; four separate reviews of venous thromboembolism (VTE) with hormone replacement therapy (involving 3 overlapping case-control studies of 20 case-control studies analysed) ^1-3^, three reviews of venous thromboembolism (VTE) with oral contraceptives (6 of 14 cohort studies, and 2 of 24 case-control studies)^2, 4, 5^, three reviews of gastrointestinal complications with NSAIDS (involving 6 of 24 cohort studies and 8 of 75 case-control studies)^6-8^ and 2 reviews of stroke with oral contraceptives (involving 1 of 7 cohort studies, and 9 of 30 case-control studies).^9, 10^

For the sensitivity analysis, the older meta-analyses pertaining to venous thromboembolism (VTE), gastrointestinal complications and stroke were removed so that the modest overlap can be further reduced, with only one review per specific adverse effect for the sensitivity analysis. The most recent meta-analyses Agency for Healthcare Research and Quality 2002 for venous thromboembolism (VTE) with hormone replacement therapy (HRT), Bergendal 2009 for venous thromboembolism (VTE) with oral contraceptives, Henry 2003 for gastrointestinal complications with NSAIDS, and Chan 2004 for stroke with oral contraceptives were used for analysis. This yields a ROR which is very similar to the original estimate:

- 0.94 (95% CI 0.87 - 1.02) for cohort versus case-control studies

**References**

1. Agency for Healthcare Research and Quality. *Hormone Replacement Therapy and Risk of Venous Thromboembolism*. Rockville, MD: Agency for Healthcare Research and Quality 2002.

2. Douketis JD, Ginsberg JS, Holbrook A, Crowther M, Duku EK, Burrows RF. A reevaluation of the risk for venous thromboembolism with the use of oral contraceptives and hormone replacement therapy. *Arch Intern Med.* 1997;157:1522-30.

3. Oger E, Scarabin PY. Assessment of the risk for venous thromboembolism among users of hormone replacement therapy. *Drugs Aging* 1999;14:55-61.

4. Koster T, Small RA, Rosendaal FR, Helmerhorst FM. Oral contraceptives and venous thromboembolism: a quantitative discussion of the uncertainties. *J Intern Med* 1995;238:31-7.

5. Bergendal A, Odlind V, Persson I, Kieler H. Limited knowledge on progestogen-only contraception and risk of venous thromboembolism. *Acta Obstet Gynecol Scand* 2009;88:261-66.

6. Bollini P, Garcia RLA, Pérez GS, Walker AM. The impact of research quality and study design on epidemiologic estimates of the effect of nonsteroidal anti-inflammatory drugs on upper gastrointestinal tract disease. *Arch Intern Med.* 1992;152:1289-95.

7. Henry D, McGettigan P. Epidemiology overview of gastrointestinal and renal toxicity of NSAIDs. *Int J Clin Pract Suppl* 2003;135:43-9.

8. Ofman JJ, MacLean CH, Straus WL, Morton SC, Berger ML, Roth EA, et al. A meta-analysis of severe upper gastrointestinal complications of non-steroidal anti-inflammatory drugs. *J Rheumatol* 2002;29:804-12.

9. Chan WS, Ray J, Wai EK, Ginsburg S, Hannah ME, Corey PN, et al. Risk of stroke in women exposed to low-dose oral contraceptives: a critical evaluation of the evidence. *Arch Intern Med* 2004;164:741-7.

10. Gillum LA, Mamidipudi SK, Johnston SC. Ischemic stroke risk with oral contraceptives: a meta-analysis. *JAMA* 2000;284:72-8.
